# Morphofunctional markers to assess disease-related pediatric malnutrition at admission and their association with length of hospital stay

**DOI:** 10.3389/fnut.2026.1741856

**Published:** 2026-06-22

**Authors:** Alda Daniela García-Guzmán, Beatriz Adriana Pinzón-Navarro, Daffne Danae Baldwin-Monroy, Salvador Ortiz-Gutiérrez, Martha Guevara-Cruz, Azalia Avila-Nava, Isabel Medina-Vera

**Affiliations:** 1 Servicio de Oncología Médica, Instituto Nacional de Pediatría, Ciudad de Mexico, Mexico; 2 Tecnologico de Monterrey, Escuela de Medicina y Ciencias de la Salud, Ciudad de México, Mexico; 3 Servicio de Gastroenterología y Nutrición Pediátrica, Instituto Nacional de Pediatría, Ciudad de Mexico, Mexico; 4 Unidad de Terapia Intensiva, Instituto Nacional de Pediatría, Ciudad de Mexico, Mexico; 5 Departamento de Fisiología de la Nutrición, Instituto Nacional de Ciencias Médicas y Nutrición Salvador Zubirán, Ciudad de Mexico, Mexico; 6 Sección de Estudios de Posgrado e Investigación, Escuela Superior de Medicina, Instituto Politécnico Nacional (IPN), Ciudad de Mexico, Mexico; 7 Hospital Regional de Alta Especialidad Península de Yucatán, Servicios de Salud del Instituto Mexicano del Seguro Social para el Bienestar (IMSS-Bienestar), Merida, Yucatán, Mexico; 8 Departamento de Metodología de la Investigación, Instituto Nacional de Pediatría, Ciudad de Mexico, Mexico

**Keywords:** body composition, disease-related malnutrition, hospital admission, pediatric, phase angle

## Abstract

**Background:**

Disease-related malnutrition (DRM) occurs due to energy imbalance caused by increased catabolism, decreased appetite, or increased energy losses. In hospitalized patients, the presence of DRM can increase morbidity, disability, and mortality, as well as delay recovery from the disease. Therefore, identifying accurate nutritional markers associated with length of stay (LOS) in pediatric patients is important.

**Objective:**

The objective of this study was to explore the association between morphofunctional markers used to evaluate DRM upon hospital admission and LOS in pediatric patients.

**Methods:**

Exploratory, single-center prospective cohort study was performed in a single center study with patients evaluated within 24–48 h of a third level pediatric hospital. Upon hospital admission, nutritional risk was determinate risk nutritional status screening, anthropometric measurements were made such as weight, height, mid-upper arm circumference (MUAC), calf circumference (CC); body composition with bioelectrical impedance analysis (BIA) and handgrip strength (HGS) was carried out. DRM was diagnosed based on body mass index-for-age z-score (BMIz) < −2 standard deviations (SD).

**Results:**

A total of 139 pediatric patients (mean age 12.1 ± 3.3 years) were included; DRM was present in 15.8%. Among patients with DRM, those who concomitantly presented low phase angle (PhA), low CC, and low HGS had a significantly longer median LOS [15 (5–27) days] than those with values above the established cutoff points [(8 (5–13) days], (*p* = 0.029).

**Conclusion:**

In pediatric patients with DRM, lower levels of morphofunctional markers (PhA, HGS, and CC) at hospital admission were associated with prolonged hospital stay. These findings, derived from a Mexican pediatric population, warrant external validation in larger and more diverse cohorts.

## Introduction

Malnutrition is a significant concern among hospitalized patients. Delayed diagnosis is associated with adverse clinical outcomes, including increased mortality, higher comorbidity, and greater hospital costs (1). The American Society for Parenteral and Enteral Nutrition (ASPEN) has defined pediatric malnutrition as “an imbalance between nutrient requirement and intake, resulting in cumulative deficits of energy, protein, or micronutrients that may negatively affect growth, development, and other relevant clinical outcomes” (2, 3). Therefore, early diagnosis of disease-related malnutrition (DRM) caused by acute or chronic conditions is essential. DRM develops due to energy imbalance. This imbalance may result from increased catabolism, decreased appetite, or increased energy losses. These mechanisms have detrimental effects on clinical outcomes (4).

Malnutrition screening tools (MSTs) have been developed to facilitate the early identification of pediatric patients at nutritional risk or with existing malnutrition at hospital admission (5). However, DRM is also characterized by functional and body composition changes driven by the inflammatory process associated with the underlying disease. Muscle depletion due to the catabolism is a key feature (6). Assessing muscle decline and fat distribution during hospitalization is clinically relevant. Chronic diseases and hospital-related immobilization contribute to the loss of muscle mass and function, as well as to alteration in body composition (7–9). Therefore, evaluating body composition is particularly important in this population.

In recent years, bioelectrical impedance analysis (BIA) has gained significant attention in pediatric populations as a non-invasive, easy to use method for monitoring growth, body composition, and nutritional status. BIA allows to measure body compartments such as fat-free mass (FFM), fat mass (FM), total body water (TBW), and body cell mass (BCM), among others (10, 11). Recent studies have documented that other parameters derived from the BIA such as phase angle (PhA) have emerged as important markers for functional and nutritional status. PhA is calculated from resistance and reactance at 50-kHz frequency, and it is a marker of cell integrity, size, and function, showing correlation with various clinical outcomes in both pediatric and adult populations (12, 13). Few studies have evaluated the association between PhA with malnutrition in pediatric patients at hospital admission (14), highlighting the need for further research. Other functional parameters include handgrip strength (HGS) and calf circumference (CC). HGS has been associated with reduced mortality and length of stay (LOS), making it a valuable indicator of muscle function and mass (15–17). CC is an anthropometric measurement linked to FFM that has been associated with malnutrition risk, morbidity, and mortality rates in hospitalized adults (18, 19).

Although morphofunctional markers such as PhA, CC, and HGS have demonstrated growing potential for assessing cellular integrity, muscle mass, and neuromuscular function, their clinical utility in hospitalized pediatric populations remains insufficiently defined. The available pediatric evidence is limited and heterogeneous, constraining our understanding of their capacity to support early identification of nutritional deterioration and to predict clinically relevant outcomes, including length of hospital stay. This lack of clarity has hindered the systematic incorporation of these markers despite being accessible, non-invasive, and highly sensitive to physiological change into routine pediatric nutritional assessment. In this context, the present study aims to deepen understanding of their clinical relevance by examining their association with disease-related malnutrition and hospital length of stay. By elucidating their potential applicability and clinical significance, this study seeks to underscore the value of a morphofunctional approach for strengthening early risk identification, improving clinical stratification, and optimizing care in hospitalized pediatric patients.

## Participants and methods

### Study design

An exploratory, single-center prospective cohort study was conducted in a third level pediatric hospital in Mexico City with a sample of 181 pediatric. Children aged 6–18 years were included if the evaluation was performed within 24–48 h of admission in the medical oncology and gastroenterology wards. Nutritional assessment included screening the risk malnutrition using STRONGkids MST, along with anthropometric measurements, BIA, and HGS. Patients with amputations or those who were unable to undergo BIA assessment were excluded from the study.

The sample size was determined based on the number of eligible patients available during the study period who met the predefined inclusion criteria. Due to the specific characteristics of the study population and the limited availability of participants, a relatively small sample was included. This study should therefore be considered exploratory, and the findings should be interpreted in the context of this sample size.

### Clinical and anthropometric markers

Clinical and demographic characteristics, including age, sex, and diagnosis, were collected. Anthropometric assessment included measurement of weight, height, mid-upper arm circumference (MUAC), tricipital skinfold (TSF), waist, neck and calf circumferences, knee height (KH), and half-span.

Participants were weighted on a calibrated digital scale (SECA 813; Seca GmbH&Co., Hamburg, Germany), and height was measured with an ultrasonic stadiometer (InLab S50; InBody Co., Seoul, Korea), MUAC was measured with the arms by the side of the body, and the measurement tape was put on the half between the measurement of the acromion and the olecranon; TSF was measured with a skinfold caliper (AnthroFlex; NutriActiva, Minnesota, United States); the midpoint of the upper arm was identified from the posterior aspect of the subject with the arm flexed at a 90° angle and the palm facing upward. The lateral tip of the acromion and the most distal point of the olecranon were located, and the distance between these two landmarks was measured. The midpoint of this distance was then marked. With the arm relaxed and hanging freely, a skinfold was grasped parallel to the longitudinal axis of the arm. The caliper was applied perpendicular to the fold, approximately 1 cm from the marked midpoint. Measurements were recorded to the nearest 0.1 cm and performed in triplicate; waist circumference was measured with the arms crossed over the chest, the measure was taken between the lower edge of the 10th rib and the iliac crest, and neck circumference was measured at the midpoint of the neck, below laryngeal prominence, and above the clavicle at the end of normal expiration. All circumferences were measured using a non-stretchable anthropometric measuring tape (SECA 201; Seca GmbH&Co., Hamburg, Germany). CC was measured at the level of the greatest circumference of the calf; KH was determined while the participants were lying down in a supine position, with legs positioned at a 90-degree angle, and the tape was placed perpendicular to the bed, aligned with the lateral side of knee joint. Measurement was taken from the highest point of the lateral condyle of the femur to the heel (20), and half-span was taken from the acromion to the middle finger distal phalanx. The z score was used in MUAC, TSF, height-for-age z score (HAz), and body mass index-for-age z score (BMIz). The other determinations were absolute measurements. In this study, disease-related malnutrition was operationally defined using BMI-for-age z-score, with a cutoff value of < −2 SD, based on the anthropometric indicators proposed in the consensus statement for pediatric malnutrition (21). Risk of malnutrition was assessed with the validated Spanish Version of the STRONGkids MST (22).

### Phase angle and bioelectrical impedance parameters

BIA assessments were performed using a multifrequency bioimpedance analyzer (InBody S10®, InBody Co., Ltd., Seoul, Korea); with the standard technique, BIA’s internal equation was used. The participants were measurements with minimally clothed and free from any equipment or metal accessories that could interfere with the impedance analysis. Measurements were taken with the participants in a supine position with arms separated from trunk by about 30 degrees and legs separated by about 45 degrees, no contact with metal frame of bed, and at ambient temperature in the room. The patients rested for 5 min and were not allowed to eat or make any big physical effort in the preceding 8 h, and not drink in the preceding 3 h. The fasting and fluid restriction periods were established to reduce the influence of recent intake on hydration status and impedance-derived measurements, following previously published clinical BIA recommendations for standardized assessments (23, 24). Body weight and height were entered into the device prior to analysis. The skin areas for electrode placement were cleaned with alcohol and with an electroconductive wet wipe for impedance equipment, the electrodes were placed on both hands and feet according to the manufacturer’s instructions (InBody Co). The electrodes were kept in a sealed bag to protect from heat. The device was calibrated before use with a circuit of known impedance, as per manufacturer’s guidelines. A standardized healthcare professional performed the study using the same device to avoid interobserver and inter-device variability. PhA at 50 kHz was recorded and automatically calculated using the following formula: [Arc tangent (Xc/R)] x (180/*π*) (25). The skeletal muscle index (SMI) was calculated by dividing skeletal muscle mass (kg) by the square of the height (m (1)), and impedance ratio was calculated as the quotient of Z at 250 kHz divided by Z at 5 kHz.

### Handgrip strength evaluation

HGS was assessed using a Lafayette hydraulic hand dynamometer (Jamar model J00105 Lafayette Instrument Company, United States. 90 kg capacity and 727 g weight). Measurements were obtained from the dominant hand in triplicate, and the highest measurement was recorded. Grip strength was measured with patients in standing, with legs straight and weight bearing balanced on both feet, feet shoulder-width apart. The shoulder adducted and neutrally rotated, elbow flexed to 90°, forearm in neutral position, and wrist between 0° and 30° of dorsiflexion and between 0° and 15° of ulnar deviation (26).

### Length of hospital stay

LOS was calculated by counting the days from hospital admission to discharge, expressed in days.

### Statistical analysis

Continuous variables were expressed as mean ± standard deviation or medians and percentiles (25th–75th) depending on distribution variable; dichotomous variables were expressed as frequencies and percentages. The Kolmogorov–Smirnov test was used to evaluate the normality of the variables. Differences between groups were assessed using X (1), independent sample *t*-test or Mann–Whitney U test. Cutoff points for BIA-derived parameters, CC, and HGS were obtained by receiver operator characteristic (ROC) curve. A correlation analysis with Pearson’s correlation coefficient calculated for anthropometric variables and the BIA variables was performed, and it was visualized using a heatmap. Associations between PhA, CC, and HGS with DRM and LOS were evaluated using logistic regression models while controlling for potential confounding factors (age and sex). To evaluate length of stay in the hospital stratified by levels of PhA, HGS and CC Kaplan–Meier curve were used considering hospital discharge as the event of interest. No in-hospital mortality events occurred during the study period; therefore, competing risk analysis was not required. The significance value of P was < 0.05. Analysis was performed with software SPSS version 25.0 (SPSS Inc., Chicago, IL, United States) and GraphPad Prism version 9.0 (Graph Pad Software, Inc.).

### Ethical statement

The protocol was approved by the Instituto Nacional de Pediatría Research and Ethics Committees with number 2019/059, officially registered at the Office for Human Research Protections of the NIH^1^ with numbers IRB00013674 and IRB00013675. All the subject’s information was handled confidentially. Each participant and parents or primary caregivers signed a written informed consent, respectively, before enrollment.

## Results

**Figure 1:**
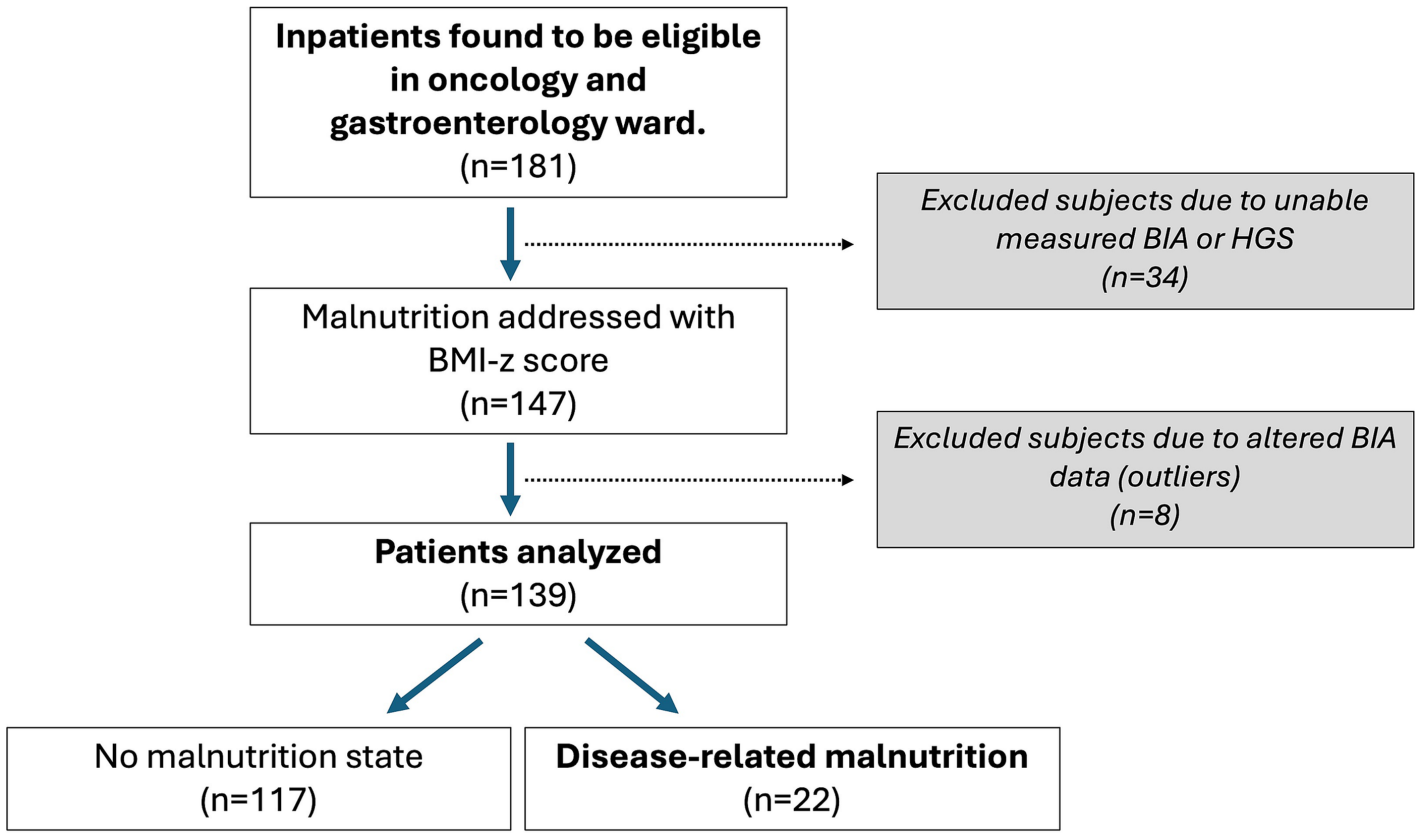
Flowchart of the patients evaluated.

Of the 181 initially eligible patients, 34 were excluded from the analysis because BIA or HGS could not be measured; the reasons for exclusion included the following: (1) clinical conditions contraindicating BIA assessment (e.g., presence of medical devices), (2) physical or clinical instability preventing adequate positioning or cooperation, and (3) inability to perform the HGS test due to limited physical capacity; and of the 147 measured, 8 patients were excluded from the analysis because they had negative or outlier BIA values (Figure 1).

### Characteristics of the participants

**Figure 2:**
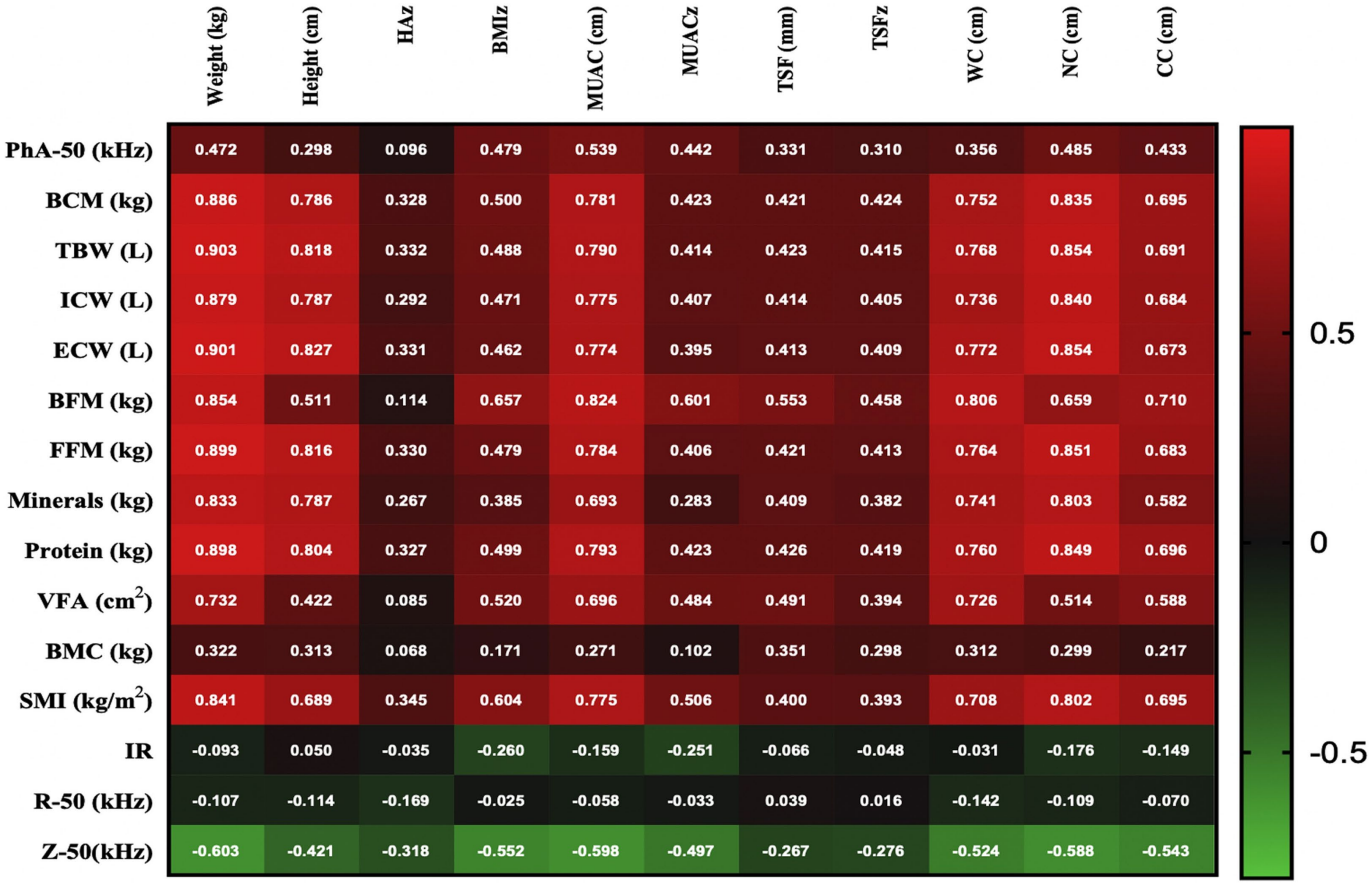
Heatmap of correlation between anthropometric indicators and BIA indicators. Red color indicates positive correlations and the green color negative correlations. HAz, height-to-age z score; BMIz, body mass index-for-age, z score; MUAC, mid-upper arm circumference; TSF, tricipital skinfold; WC, waist circumference; NC, neck circumference; CC, calf circumference; PhA-50: phase angle at 50 kHz; BCM, body cell mass; TBW, Total body water; ICW, Intracellular water; ECW, Extracellular water; BFM, Body fat mass; FFM, fat free mass; VFA, visceral fat area; BMC, bone mineral content; SMI, skeletal muscle index; IR, impedance ratio; R-50(kHz): resistance at 50 kHz; Z-50(kHz): impedance at 50 kHz. Statistical analysis of Pearson’s correlations captured in a heatmap.

Among139 patients included in the study and according to the hospitalization settings, the mean age was 12.1 ± 3.3 years, and the mean height and weight were 148 ± 19 cm and 44.7 ± 18.6 kg, respectively. In total, 60% were male patients (*n* = 83). According to the nutritional risk evaluation with STRONGkids MST, 20.9% (*n* = 29) were high risk of malnutrition, 75.5% (n = 105) were medium risk, and only 3.6% (*n* = 5) were low risk. Demographic characteristics and anthropometric measurements stratified by sex are shown in Table 1. Significant differences between sexes were observed in TSF (*p* = 0.043), neck circumference (*p* < 0.0001), KH (*p* = 0.019), and half span (*p* = 0.003). Correlation analysis between anthropometric indicators and BIA indicators is shown in a heatmap. Strong positive correlations were observed between TBW and body weight (*r* = 0.903, *p* < 0.05), and FM and waist circumference (*r* = 0.806, *p* < 0.05); on the contrary, we found negative correlations with impedance at 50 kHz (Z-50 kHz) and MUAC (*r* = −0.598, *p* < 0.05) and Z-50 kHz and CC (*r* = 0.543, *p* < 0.05; Figure 2). As shown in Supplementary Table 1, patients from the oncology and gastroenterology wards presented similar anthropometric and body composition characteristics, with no statically significant differences observed between groups for most variables. However, body fat percentage was significantly higher in patients from the gastroenterology ward than those from the oncology ward (28 ± 13% vs. 22.6 ± 10%, *p* = 0.032).

**Table 1:** Characteristics of assessed patients analyzed by sex upon hospital admission.

Variables	Total *n* = 139	Female *n* = 56	Male *n* = 83	*p*-value
Age, *y*	12.1 ± 3.3	12 ± 3.3	12.2 ± 3.2	0.752
Height, *cm*	148 ± 19	144 ± 14.8	150 ± 21.0	0.073
Weight, *kg*	44.7 ± 18.6	41.4 ± 15.7	47 ± 20.0	0.080
Anthropometric measurements				
MUAC, *cm*	22.47 ± 5.3	22.3 ± 4.9	22.6 ± 5.5	0.697
HA, *z-score*	−0.34 ± 1.2	−0.57 ± 1.1	−0.19 ± 1.3	0.080
MUAC, *z-score*	−0.86 ± 1.7	−0.72 ± 1.6	−0.96 ± 1.8	0.421
TSF, *mm*	12.3 ± 5.3	13.4 ± 5.2	11.6 ± 5.3	0.043
TSF, *z-score*	0.07 ± 0.98	−0.14 ± 1	0.22 ± 0.93	0.35
Waist circumference, *cm*	71.4 ± 14	69.6 ± 13.6	72.6 ± 14.2	0.236
Neck circumference, *cm*	31.7 ± 4.2	29.9 ± 3.14	32.8 ± 4.4	< 0.0001
CC, *cm*	27.9 ± 6.6	27.6 ± 6.9	28.1 ± 6.3	0.651
Knee height, *cm*	42.1 ± 6.1	40.5 ± 5.7	43.2 ± 6.2	0.019
Half span, *cm*	73.9 ± 11.2	70 ± 10.6	76.5 ± 10.8	0.003
BMIz, *z-score*	−0.11 ± 1.7	−0.13 ± 1.5	−0.09 ± 1.74	0.902

CC, Calf circumference; BMIz, Body mass index-for-age, z-score; HAz, Height-for-age, z-score; MUAC, Mid-upper arm circumference; TSF, Tricipital skinfold; y, Years. Statistical analysis to compare between groups was independent samples *t*-test.

### Anthropometric variables, body composition, and handgrip strength in relation to disease-related malnutrition

**Figure 3:**
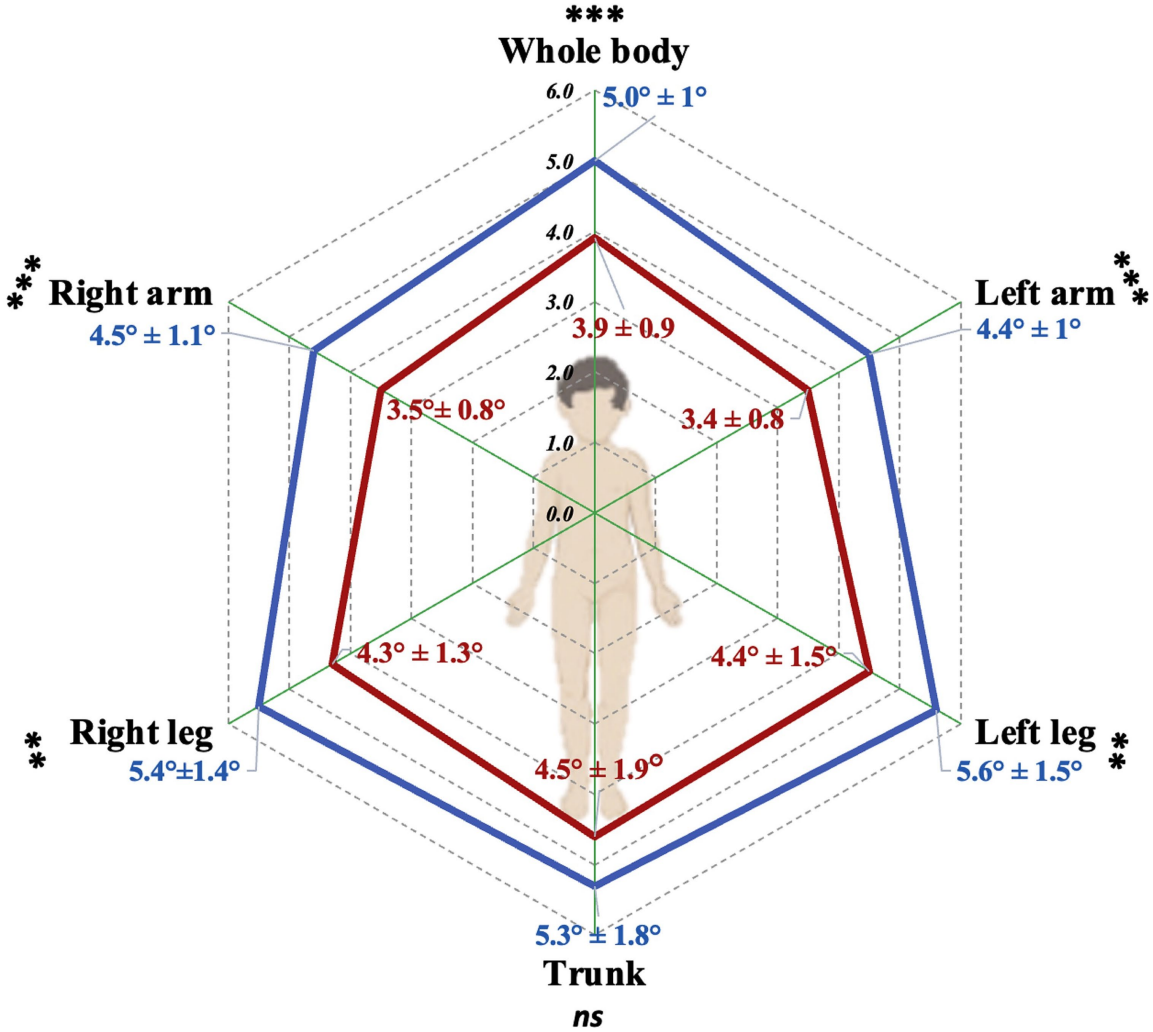
Phase angle between DRM and without malnutrition group. Blue line is no malnutrition state (*n* = 117), and red line is DRM group (*n* = 22). DRM: disease-related malnutrition according to z-score. *p*-value: ** *p* < 0.001, *** *p* < 0.0001, ns: no significant. Statistical analysis to compare between groups was independent samples *t*-test.

DRM was present in 15.8% (*n* = 22) of the patients evaluated. Anthropometric measurements, body composition, and HGS between DRM and non-malnutrition state were addressed. As expected, patients with DRM had significantly lower anthropometric measurements in all evaluations except KH and half-span measurement. Regarding body composition measured by BIA, the difference between DRM and non-malnutrition groups was significantly lower except for bone mineral content (*p* = 0.210); in addition, children with DRM showed less HGS in dominant arm (*p* < 0.0001; Table 2). When we compared the total PhA between groups, lower total PhA was observed in the DRM group (3.9 ± 0.9 vs. 5 ± 1, *p* < 0.0001); in addition, the PhA for extremities was lower and statistically significant, except for trunk PhA although it was lower in the DRM group it was not statistically significant (4.5° ± 1.9° vs. 5.3° ± 1.8°, *p* = 0.084; Figure 3).

**Table 2:** Anthropometric variables, body composition, and handgrip strength in disease-related malnutrition according to BMIz.

Parameters	NMS *n* = 117	DRM *n* = 22	*p*
Demographics			
Age, *y*	12 ± 3.2	11.5 ± 3.5	0.084
Anthropometric variables			
Height, *cm*	149 ± 18.5	143 ± 4.6	0.156
Weight, *kg*	47.7 ± 18.2	28.5 ± 10.1	0.0001
BMIz, *z-score*	−0.41 ± 1.2	−2.8 ± 0.77	< 0.0001
HAz, *z-score*	−0.24 ± 1.1	−0.85 ± 1.6	0.036
MUAC, *cm*	23.5 ± 5	17.2 ± 2.7	0.0001
MUAC, *z-score*	−0.43 ± 1.4	−3.2 ± 1.4	0.0001
Tricipital skinfold, *mm*	13.08 ± 5.3	8.4 ± 3	0.0001
Tricipital skinfold, *z-score*	0.21 ± 0.89	−0.64 ± 1	0.0001
Waist circumference, *cm*	73.7 ± 13.7	58.4 ± 6.5	0.0001
Neck circumference, *cm*	32.5 ± 4	28.3 ± 3.1	0.0001
Calf circumference, *cm*	28.5 ± 6.4	22.3 ± 4.3	0.0001
Knee height, *cm*	42.5 ± 5.5	40.3 ± 8.1	0.124
Half span, *cm*	71.4 ± 10.9	71.3 ± 11.8	0.227
Body composition			
Proteins, *kg*	6.5 ± 2.3	4.6 ± 1.7	< 0.0001
Minerals, *kg*	2.4 ± 0.8	1.9 ± 0.5	0.010
Bone mineral content, *kg*	2.3 ± 2.1	1.6 ± 0.4	0. 210
Body cell mass, *kg*	21.5 ± 7.7	15.4 ± 5.9	< 0.0001
Skeletal muscle mass, *kg*	17.7 ± 6.8	12 ± 5.4	< 0.0001
SMI, *kg/m^2^*	5.5 ± 3.2	3.5 ± 1.5	0.006
Fat free mass, *kg*	33.5 ± 11.4	24.4 ± 9	0.001
Fat mass, *kg*	13.5 ± 9.6	4.4 ± 3.9	< 0.0001
Body fat, *%*	25.6 ± 11.1	14.5 ± 10.2	< 0.0001
Total body water, *l*	24.6 ± 8.3	17.8 ± 6.8	< 0.0001
Intracellular water, *l*	15.1 ± 5.4	10.7 ± 4.2	0.001
Extracellular water, *l*	9.5 ± 3.1	6.9 ± 2.6	0.001
IR 250/50, *kHz*	0.90 ± 0.030	0.92 ± 0.02	0.003
Handgrip strength			
Right arm, *kg*	14.5 ± 7	9.3 ± 4.6	0.003
Left arm, *kg*	12 ± 6.3	8.5 ± 6.4	0.088
Dominant arm, *kg*	14.4 ± 6.9	7.5 ± 2.3	< 0.0001

NMS, No malnutrition state; BMIz, Body mass index-for-age, z-score; DRM, disease-related malnutrition; MUAC, Mid-upper arm circumference; SMI, Skeletal muscle mass index; IR, Impedance ratio (250/50, kHz); HAz, height-to-age, z score. Statistical analysis to compare between groups was independent samples *t*-test.

### Morphofunctional markers cutoff values to detect disease-related malnutrition in admitted patient

Optimal cutoff values for several nutritional markers, including total PhA at 50 kHz, HGS, SMI, BCM, IR (250/50 kHz), and CC were determined using ROC curve analysis. The highest area under the curve (AUC) was observed for CC, HGS, and PhA, and the one that showed the lowest AUC was BCM; cutoff points associated with DRM were generated; for CC it was <27.5 cm, for HGS it was <11.5 kg, and for PhA it was < 4.5°; with these cutoff points, it was decided to perform combined variables of these morphofunctional markers, where it was observed that they had a lower AUC than the markers alone to relate to DRM (Table 3). Furthermore, a logistic regression was performed to assess the risk of DRM and the cutoff points generated previously with the morphofunctional markers. The results showed that a low CC, low PhA, and low HGS were associated with a higher risk of malnutrition (Table 4).

**Table 3:** Optimal variable cutoff values to detect disease-related malnutrition according to BMIz in admitted patient.

Parameters	AUC (CI 95%)	*p*-value	Cutoff point	Sensitivity (%)	Specificity (%)	PPV	NPV
CC, *cm*	0.820 (0.738–0.879)	<0.0001	< 27.5 cm	96.1	60.9	2.9	0.25
HGS, *kg*	0.789 (0.722–0.857)	<0.0001	< 11.5 kg	96.5	57.4	2.48	0.07
PhA, °	0.789 (0.688–0.889)	<0.0001	< 4.5°	81.8	64.4	2.33	0.28
SMI, kg/m^2^	0.783 (0.689–0.877)	<0.0001	<4.5 kg/m^2^	81.8	66.6	2.45	0.27
IR, 250/50 *kHz*	0.752 (0.643–0.861)	0.0002	< 0.9 kHz	81.8	52.9	1.64	0.11
BCM, *kg*	0.733 (0.627–0.839)	0.0005	< 20.5 kg	81.8	53.8	1.77	0.34
Combined indicators							
PhA + CC	0.768 (0.646–0.890)	0.0004	4.5°/ 27.5 cm	80	72	2.9	0.28
PhA + SMI	0.722 (0.595–0.848)	0.0031	4.5° / 4.5 kg/m^2^	80	64	2.25	0.31
PhA+ CC + HGS	0.720 (0.580–0.861)	0.0037	4.5°/27.5 cm/11.5 kg	63	79	3.11	0.46
PhA + SMI + HGS	0.698 (0.557–0.839)	0.0089	4.5°/4.5 kg/m^2^/11.5 kg	63	76	2.69	0.49

PPV, Positive predictive value; NPV, Negative predictive value; CC, Calf circumference; HGS, Handgrip strength; PhA, Phase angle; SMI, Skeletal muscle index; IR, Impedance ratio; BCM, Body cell mass. Statistical analysis through receiver operator characteristic curve.

**Table 4:** Odds ratio (OR) and 95% confidence intervals (CIs) for variables associated with DRM according to z-score.

Variables	Raw OR		Adjusted OR*	
	OR (CI 95%)	*p*	OR (CI 95%)	*p*
50 kHz PhA, (< 4.5°)	8.3 (2.6–26.2)	<0.0001	7.8 (2.4–25)	0.001
CC, (< 27.5 cm)	9.7 (3.4–27.5)	<0.0001	13.6 (3.9–47)	< 0.0001
HGS, (<11.5 kg)	35 (4.5–268)	0.001	42 (5–347)	0.001
Impedance ratio, (< 0.9 kHz)	15 (1.9–116)	0.009	14 (1.8–113)	0.010
BCM, (< 20.5 kg)	3.4 (1.1–10.9)	0.033	3.8 (0.99–14.9)	0.051

*Adjusted by age and sex. OR, Odds ratio; PhA, Phase angle; CC, Calf circumference; HGS, Handgrip strength; BCM, Body cell mass. Logistic regression statistical analysis.

### Hospital length of stay

The study evaluated LOS, compared on the previously addressed cutoff points for various markers. Only a significant difference in LOS was found between patients with low PhA alone and those with values above the cutoff point; patients with PhA < 4.5° had a median LOS of 9 (6–19) days, whereas those with PhA > 4.5° had a median LOS of 8 (4–14) days (*p* = 0.032).

**Figure 4:**
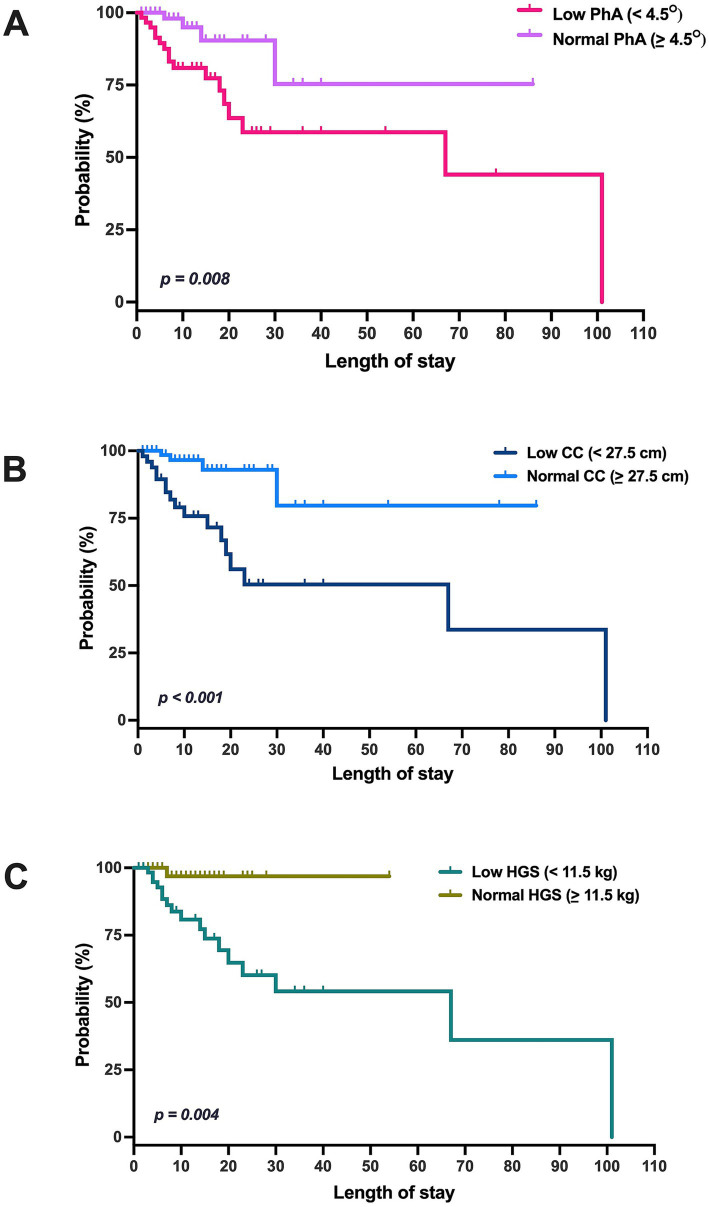
Kaplan–Meier curve for hospital stay and DRM in hospitalized patients stratified by **(A)** cut-off point for PhA < 4.5°; **(B)** cut-off point for CC < 27.5 cm; **(C)** cut-off point for HGS < 11.5 kg. Statistical analysis Kaplan–Meier curve.

In addition, a combine marker analysis was performed, integrating PhA, CC, and HGS into a single composite variable. This variable was defined as the simultaneous presence of low values in all three markers (below their respective cutoff points). Patients presenting with all three markers below the established thresholds had a median LOS of 15 (5–27) days, whereas those with values above the cutoff points showed a significantly lower median LOS of 8 (5–13) days (*p* = 0.029). Kaplan–Meier curves for hospital stay for low PhA, HGS, and CC related to DRM are illustrated in Figure 4. The log-rank test revealed significant differences between the curves (*p* < 0.0001).

## Discussion

Our study findings are consistent with previous research showing a high prevalence of malnutrition among hospitalized pediatric patients (27) which has been reported from 9 to 26.6%, and the prevalence observed in our cohort patient population was 15.8%. In DRM, pre-existing inflammatory state contributes to energy imbalance through multiple mechanisms: increased catabolism, reduced appetite, and increased energy losses. When these alterations persist alongside inadequate intake, nutritional status deteriorates further, negatively impacting outcomes (28). These consistent findings underscore the importance of routine nutritional assessment and early intervention in pediatric medical-nutritional therapy to prevent and manage malnutrition.

In this study, cutoff values for several morphofunctional markers associated with DRM including PhA, HGS, SMI, BCM, IR, and CC were determined using ROC curve analysis. Among these, PhA, HGS, and CC demonstrated good diagnostic performance for identifying DRM in our cohort of hospitalized pediatric patients. However, it is important to emphasize that these thresholds were derived from a specific Mexican pediatric population and should be interpreted within this context. External validation in larger and ethnically diverse pediatric populations is required before broader clinical application.

PhA is considered as a physiological indicator of cell membrane integrity and reflects both the quantity and quality of soft tissues. Low PhA values have been associated with impaired cellular health, poorer prognosis, and increased morbidity and mortality. We observed in our study that the hospitalized pediatric with DRM showed a low PhA (3.9 ± 0.9°) and the cutoff of PhA related to DRM was <4.5° (AUC = 0.789); other studies have showed similar levels of PhA-related malnutrition but in other conditions, for example, Marino et al. (29), in children with primary ciliary dyskinesia (7.0 ± 5.2 y) observed low PhA (4.3 ± 0.4°) when they analyzed moderate malnutrition with z score of FFM (< −2 z-score); in addition, Girma et al. (30) reported low PhA in children (28 ± 15 months) with severe acute malnutrition with PhA of 2.2 ± 0.7°. PhA has also been proposed as a prognostic marker in hospitalized pediatric patients, with low values associated with increased mortality risk (RR: 1.51; 95%CI (1.22–1.88)) and a higher complication risk (OR: 8.17; 95%CI (2.44–27.4)) (31).

CC was evaluated as an indirect indicator of muscle mass depletion, and previous studies have related CC with muscle mass in hospitalized individuals; however, most studies have been carried out in the geriatric population (32). We identified a CC cutoff of <27.5 cm (AUC = 0.820) associated with DRM in our population. This value is comparable to that reported by Ferretti et al. (5), who used BMI-for-age z-score as the reference standard for malnutrition. In their study, calf circumference (CC) demonstrated good diagnostic performance across sex and age groups. Among females, the optimal CC cutoff was <24.4 cm for those aged 10–12 years (AUC = 0.982) and <29.1 cm for those aged 13–15 years (AUC = 0.854). Among males, the corresponding cutoff values were <25.3 cm for those aged 10–12 years (AUC = 0.973) and <28.2 cm for those aged 13–15 years (AUC = 0.873). CC is a simple, low cost, and easily applicable anthropometric measurement that may help identify muscle wasting in pediatric hospital setting.

HSG was assessed as a marker of muscle function and undernutrition. We observed a cutoff related to DRM of HGS < 11.5 kg in our population; in addition, HGS was evaluated as a measurement of muscle function; it is a reliable indicator of undernutrition. Jensen et al. (33) observed a similar cutoff in hospitalized patients (6–14 years) of 12.4 ± 0.37 kg (mean ± SE), and HGS was associated with age, height, and MUAC z scores. Additionally, Silva et al. (34) showed that lower HGS may be a potential marker of undernutrition in hospitalized pediatric patients; in his multivariate analysis, sex, age, height, and BMI z scores explained 67.1% of HGS at hospital admission. Furthermore, Suominen et al. (35) reported that lower grip strength was associated with reduced muscle mass and a higher prevalence of frailty later in life highlighting the importance of early nutritional and functional interventions. Additionally, in our cohort, low PhA alone values were associated with a modest but statistically significant increase in LOS (9 (6–19) days vs. 8 (4–14) days (*p* = 0.032). Although the absolute difference was small, this finding suggests a potential relationship between cellular integrity and clinical outcomes in this hospitalized pediatric population and should be interpreted within the context of the study’s sample size and population characteristics. However, similar data had showed in pediatric population with nutritional deterioration (reduction in body mass index ≥ 0.25 Z-score) in hospitalized children (4 (2 to 6) days vs. 5 (3 to 8) days, *p* = 0.002) (36). In addition, when considering a multiple morphofunctional marker analysis with PhA, CC, and HGS, we found significant differences in LOS between patients with low and normal values of these markers (15 (5–27) days vs. 8 (5–13) days; *p* = 0.029) increasing the days of hospital stay in the affected children. Studies in critically pediatric patients showed that a low PhA (> 2.8°) was more likely to remain in the intensive care unit (ICU) compared to those who had a PhA > 2.8° (HR: 1.64 (95% CI. (1.09–2.47); *p* = 0.003), once adjusted for sex, age, and the Pediatric Index of Mortality; interestingly, when the ICU stay of those children with MUAC < 5th percentile was analyzed, it was not observed (HR: 1.30 (95% CI. (0.98–1.71); *p* = 0.063) (37). In line with our study, Yates et al. (37) observed that standardized PhA was associated with longer hospital stays in adult patients with acute hematologic malignancies, while Sasse et al. (38) have already highlighted the importance of early and individualized nutrition therapy in oncologic patients with solid tumors, a major percentage from our studied sample. Consistent with previous studies, our findings emphasize the importance of early identification of malnutrition in pediatric hospitalized patients to improve outcomes and reduce LOS (39, 40).

The rationale for performing the combined analysis of PhA, HGS, and CC was based on their complementary physiological dimensions. PhA reflects cellular integrity and body composition (41), HGS assesses muscle function (42), and CC serves as a proxy for muscle mass (43). Therefore, their combined evaluation provides a more comprehensive assessment of muscle health and nutritional status than any single parameter alone. Although the combined model presented a slightly lower AUC compared to some isolated markers, we considered it clinically relevant because it integrates structural and functional components of muscle status, potentially improving risk stratification in heterogeneous pediatric populations. Additionally, the combined approach may enhance clinical applicability by reducing reliance on a single measurement that could be influenced by acute clinical conditions.

Evidence regarding cutoff points for morphological markers used to assess nutritional status in pediatric populations remains limited. A recent systematic review (38) identified several studies that proposed threshold values using ROC curve analysis in different pediatric clinical populations. In this review, a standardized PhA ≤ 0 SD was associated with malnutrition in children undergoing hematopoietic stem cell transplantation, whereas in adolescents with anorexia nervosa a value close to 4.9° was suggested to identify severe malnutrition. However, the review highlighted substantial heterogeneity in the populations studied, the criteria used to define malnutrition, and the methods used to report PhA, which currently prevents the establishment of universal cutoff values for pediatric populations. Similarly, HGS has been proposed as a functional marker of nutritional status in hospitalized pediatric patients (44). In one study, ROC curve analysis identified HGS z-score cutoff points of −0.81 (age-adjusted) and −1.2 (height-adjusted) to detect a high risk of malnutrition, with AUC values close to 0.7. In addition, calf circumference (CC) has also been explored as a simple anthropometric indicator of nutritional status, with studies reporting age- and sex-specific CC cutoff points associated with malnutrition in children and adolescents with cancer using ROC analysis. Lower CC values were associated with a higher risk of malnutrition. Nevertheless, further research is required to validate these indicators and establish standardized cutoff values across different pediatric clinical settings (30).

Our results suggest that several markers, including PhA, HGS, and CC, can be valuable tools in identifying DRM in this population. However, our study has certain limitations. First, the relatively small sample size may limit the statistical power of the analyses and reduce the generalizability of the findings. Therefore, the results should be interpreted with caution and considered preliminary and hypothesis generating. Future studies including larger and more diverse population are needed to confirm these findings and strengthen the external validity of the results. Second, sex-stratified cutoff points or z-scores were not used for PhA and HGS. Biological differences related to sex and pubertal development are known to influence body composition, muscle mass, and muscle strength in pediatric populations. In addition, the study population included patients with heterogeneous clinical conditions that may also influence LOS. Therefore, future studies with larger and more homogeneous cohorts should consider adjusting for these factors to better understand the relationship between nutritional and morphological markers and hospitalization outcomes.

Other limitation is that we only use a BMIz of less than 2 SD to diagnose DRM, which is a widely used criterion for diagnosing moderate malnutrition in children, but it has several significant disadvantages that may limit its usefulness as a sole indicator. Its exclusive use can lead to underdiagnosis and the omission of children at risk. Therefore, future studies will take this into account. Using only one criterion may leave children with a similar risk of mortality undiagnosed. The good diagnostic performance of these markers highlights their potential utility in clinical practice for the early detection of DRM and implementation of timely interventions to improve patient outcomes and long-term functionality. The inclusion of these markers in routine nutritional assessment protocols can aid healthcare providers in identifying patients who may require intensified nutritional support and care.

## Conclusion

Our study contributes to the growing body of literature supporting the use of PhA, HGS, and CC as morphofunctional markers for identifying DRM in hospitalized pediatric patients. These markers may be considered as complementary tools in routine clinical practice for early nutritional risk stratification. In this cohort, pediatric patients with low PhA (< 4.5°), low HGS (<11.5), or low CC (<27.5 cm) showed a higher likelihood of prolonged hospital stay. However, it is important to emphasize that these cutoff values were derived from a specific hospitalized Mexican pediatric population and should be interpreted with caution. External validation in larger and more diverse pediatric populations is required before broader clinical implementation. Further studies are needed to evaluate the impact of targeted nutritional interventions on these morphofunctional markers and their potential role in improving clinical outcomes.

## Data Availability

The raw data supporting the conclusions of this article will be made available by the authors, without undue reservation.

## Footnotes

1
http://ohrp.cit.nih.gov/search/search.aspx

## Glossary

ASPEN: American Society for Parenteral and Enteral Nutrition
AUC: Area under the curve
BCM: Body cell mass
BIA: Bioelectrical impedance analysis
BMIz: Body mass index-for-age, z score
CC: Calf circumference
DRM: Disease-related malnutrition
ESGPHAN: European Society for Pediatric Gastroenterology Hepatology and Nutrition
FFM: Fat-free mass
FM: Fat mass
HAz: Height-for-age, z score
HGS: Handgrip strength
ICU: Intensive care unit
IR: Impedance ratio
KH: Knee height
LOS: Length of stay
MUAC: Mid-upper arm circumference
PhA: Phase angle
ROC: Receiver operator characteristic
STRONGkids: Screening tool for risk on nutritional status and growth
SMI: Skeletal muscle index
TBW: Total body water
TSF: Tricipital skinfold
