# Supplementary material for: Morphofunctional markers to assess disease-related pediatric malnutrition at admission and their association with length of hospital stay
Source: Front Nutr. 2026 Jun 22;13:1741856. doi: 10.3389/fnut.2026.1741856 (PMC13333472; doi:10.3389/fnut.2026.1741856)
Supplement: Supplementary file 1 [file Table_1.DOCX]

| **Supplementary table 1. Characteristics of patients according to hospital wards.** | | | |
| --- | --- | --- | --- |
| **Variables** | **Oncology ward**  **n=99** | **Gastroenterology ward**  **n=40** | ***p value*** |
| Age, *y* | 12.2 ± 3.1 | 11.9 ± 3.5 | 0.657 |
| Height, *cm* | 148 ± 19 | 146 ± 18 | 0.5 |
| Weight, *kg* | 44.8 ± 17 | 44.5 ± 20 | 0.931 |
| DRM, *n (%)* | 14 (14.2) | 8 (20) | 0.324 |
| **Anthropometric measurements** | | | |
| MUAC, *cm* | 22.4 ± 4.7 | 22.8 ± 5.9 | 0.668 |
| HA, *z-score* | -0.33 ± 1.2 | -0.39 ± 1.2 | 0.804 |
| MUAC, *z-score* | -1 ± 1.6 | -0.5 ± 2 | 0.145 |
| BMIz, *z-score* | -0.13 ± 1.5 | -0.08 ± 1.8 | 0.870 |
| Calf Circumference, *cm* | 27.6 ± 6.6 | 28.5 ± 6.3 | 0.476 |
| **Body Composition** | | | |
| PhA, ° | 4.8 ± 1.1 | 4.7 ± 0.9 | 0.436 |
| SMI, *kg/m^2^* | 5 ± 1.6 | 4.6 ± 1.7 | 0.155 |
| Fat free mass, *kg* | 32.9 ± 11 | 30.1 ± 11 | 0.212 |
| Body Fat, *%* | 22.6 ± 10 | 28 ± 13 | 0.032 |
| IR 250/50, *kHz* | 0.90 ± .02 | 0.90 ± .03 | 0.823 |
| Dominant arm, *kg* | 13.4 ± 7 | 13.7 ± 7 | 0.848 |
| **DRM**: Disease related malnutrition; **LOS**: length of stay; **MUAC**: mid-upper arm circumference; **HA:** height-for-age, z-score; **y**: years; **BMIz**: body mass index-for-age, z-score; **PhA**: Phase angle; **SMI:** skeletal muscle mass index, **IR:** impedance ratio (250/50, *kHz*). Statistical analysis to compare between groups was independent samples *t* test. | | | |
